# Supplementary material for: Soil pH as an external filter shaping stink bug–Burkholderia gut symbiosis
Source: Microbiome. 2026 May 7;14:129. doi: 10.1186/s40168-026-02402-z (PMC13151246; doi:10.1186/s40168-026-02402-z)
Supplement: Supplementary file 2 — Additional file 1. [file 40168_2026_2402_MOESM1_ESM.pdf]

Supplementary information

Soil pH as an external filter shaping stink bug–*Burkholderia* gut symbiosis

Hideomi Itoh[1,*], Hiroyuki Shimoji[2], Daisuke Nakane[3], Seonghan Jang[4], Yoshitomo Kikuchi[1,4,*]

[1]Biomanufacturing Process Research Center, National Institute of Advanced Industrial Science and Technology (AIST), Hokkaido Center; Sapporo, Hokkaido 062-8517, Japan.
[2]Faculty of Agriculture, University of the Ryukyus; Nishihara, Okinawa 903-0213, Japan.
[3]Graduate School of Informatics and Engineering, The University of Electro-Communications; Chofu, Tokyo 182-8585, Japan.
[4]Graduate School of Agriculture, Hokkaido University; Sapporo, Hokkaido 060-8589, Japan.

[*]Corresponding authors: Hideomi Itoh (hideomi-itou@aist.go.jp) and Yoshitomo Kikuchi (y-kikuchi@aist.go.jp)

Contents:

Figures S1–S11

Tables S1–S8

Movies S1 and S2

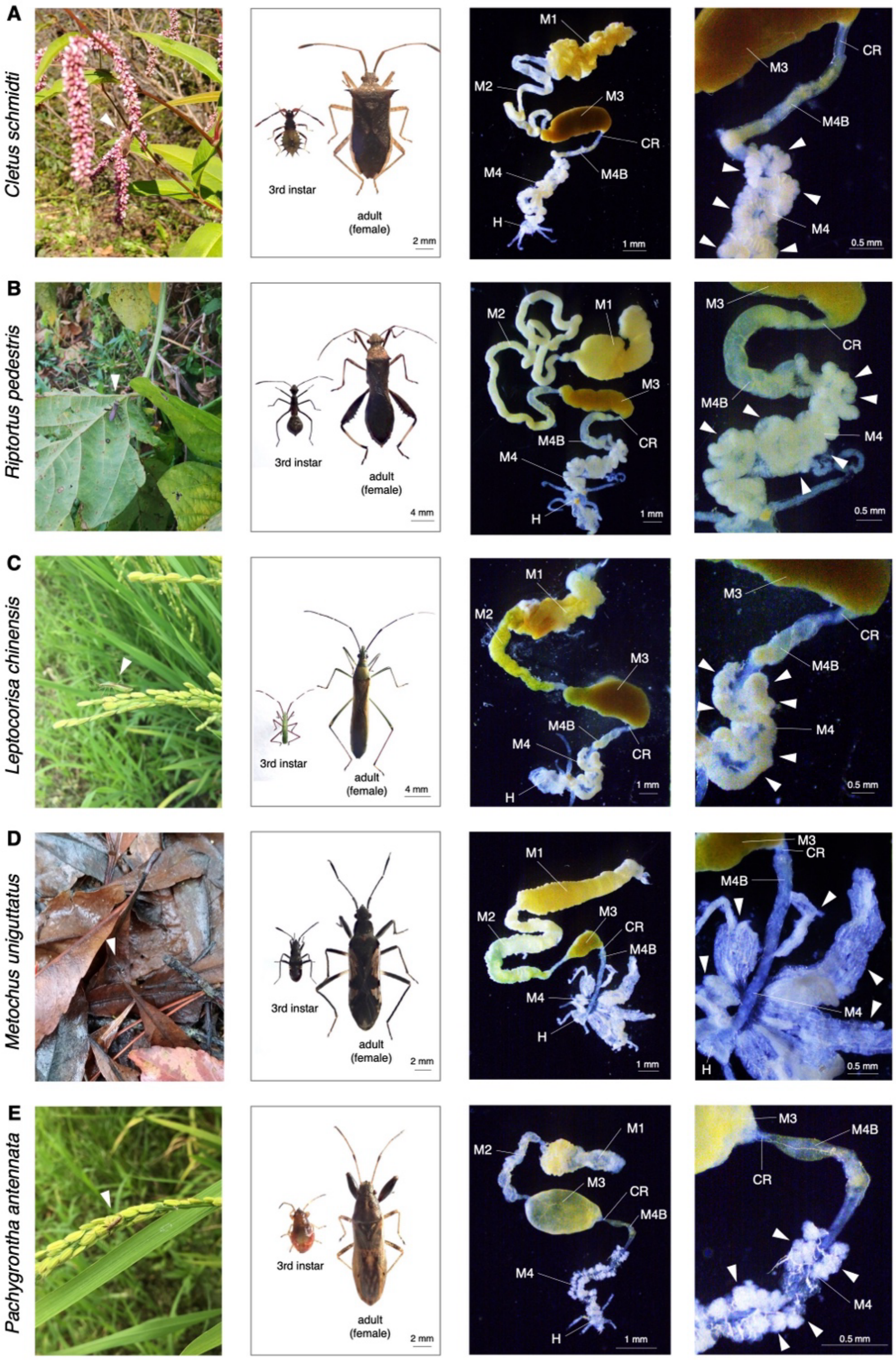

Fig. S1. Stink bug species used in this study in addition to *C. punctiger*. The first column displays ecological photographs of each insect species in their natural habitats: (A) *C. schmidti* on the flowers of *Persicaria lapathifolia*; (B) *R.pedestris* on the leaf of *Glycine max*; (C) *L. chinensis* feeding on grains of *Oryza sativa*; (D) *M.uniguttatus* moving on the ground covered with dead leaves; (E) *P. antennata* feeding on grains of *Oryza sativa*. The second, third, and fourth columns depict images of the 3rd instar nymphs and adult females of laboratory-maintained insects, the whole gut, and magnified CR, M4B and M4 regions, respectively. Abbreviations: M1, midgut first section; M2, midgut second section; M3, midgut third section; CR, constricted region; M4B, midgut fourth section with bulb; M4, midgut fourth section with crypts or tubes (symbiotic organ); H, hindgut. Closed triangles indicate crypts and tubes in M4.

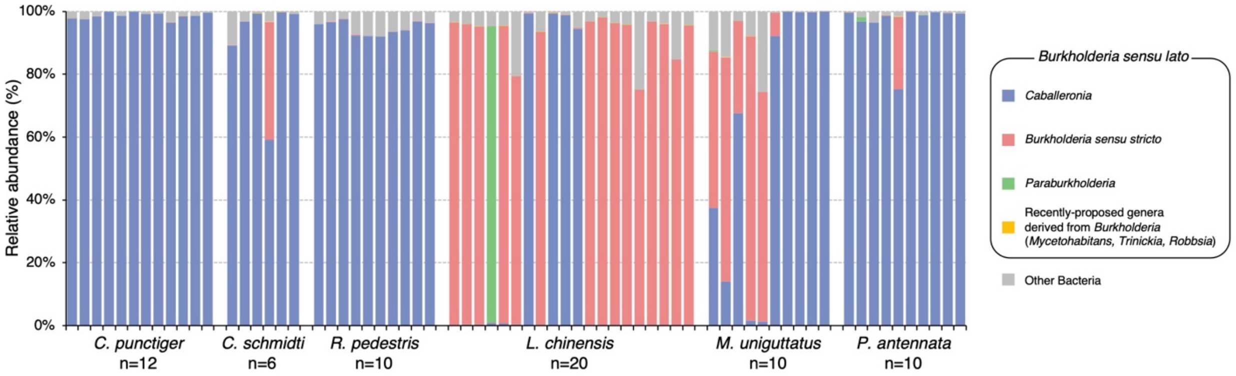

Fig. S2. Community structure of the gut microbiome in wild populations of stink bug species, estimated by PCR amplicon sequencing analysis of the bacterial 16S rRNA gene. Detailed information on the insect samples is provided in Table S2. *Caballeronia, Paraburkholderia*, *Mycetohabitans*, *Trinickia*, and *Robbsia* are derivative genera of *Burkholderia* and collectively referred to as *Burkholderia sensu lato* (Mullins and Mahenthiralingam, 2021).

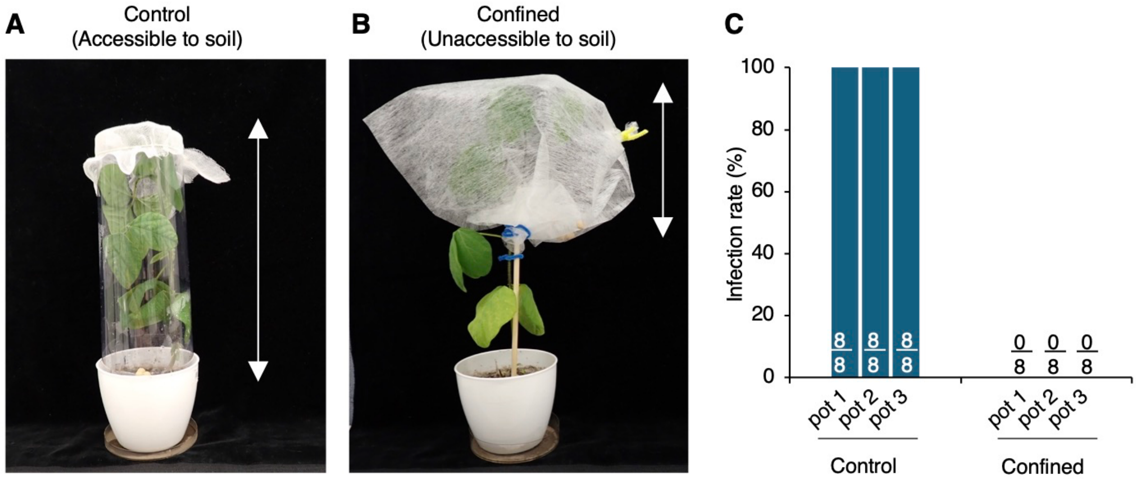

Fig. S3. Infection experiment conducted in the presence of plants. (A) and (B) Soybean pots used for the experiment. Soybean seedlings were grown in pots with soil S32. The bidirectional arrow indicates the movable range of insects (*R. pedestris*), showing that insects can access the soil in pots under control conditions (A) but cannot under confined conditions (B). (C) Infection rate of insects with *Burkholderia* after 10 days of rearing in each pot. The experiments were conducted in triplicate pots. Numbers on the bars indicate “number of positive insect samples/total number of examined insect samples.”

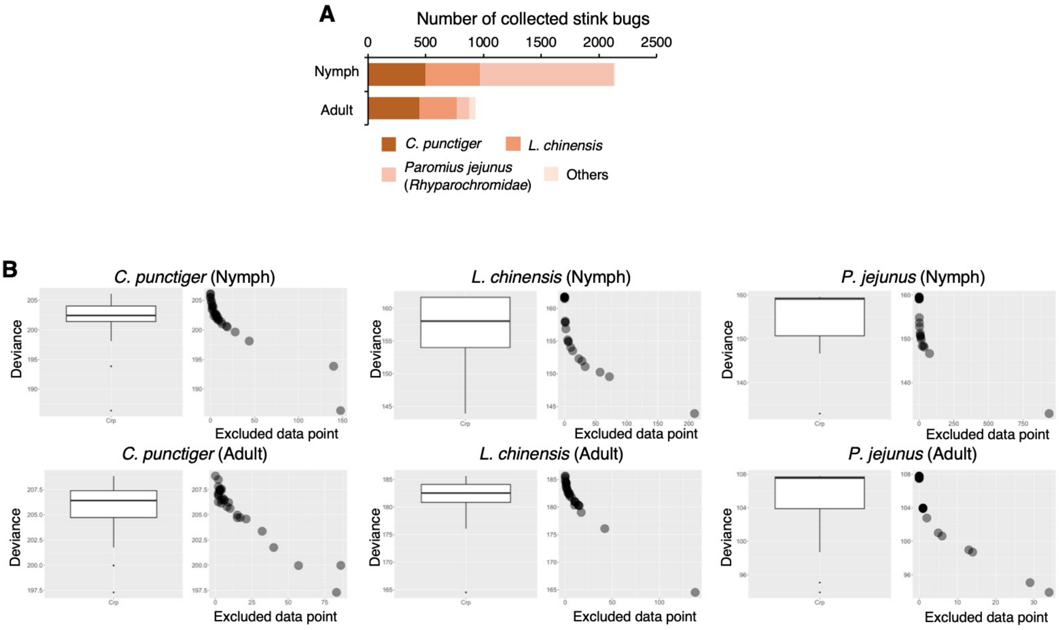

Fig. S4. Summary and statistical analysis of the field study. (A) Species composition of all collected stink bug nymphs and adults. Detailed results are provided in Table S4. (B) Sensitivity analysis of stink bug abundance in relation to soil pH. A GLM with a negative binomial distribution evaluated the relationship between soil pH and the number of stink bug species. Submodels excluding one data point at a time identified influential points by comparing the model deviance.

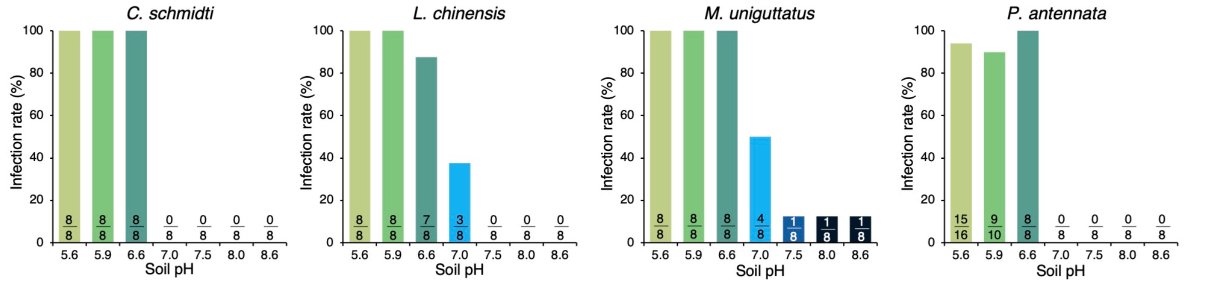

Fig. S5. pH preferences for symbiosis establishment in various stink bug species. Infection experiments were performed using seven soils with pH ranging from 5.6 to 8.6, S32 (pH 5.6), S33 (pH5.9), S34 (pH 6.6), S51 (pH7.0), S35 (pH 7.5), S36 (pH 8.0), and S37 (pH 8.6) (Table S5), and four stink bug species: *C. schmidti*, *L. chinensis*, *M. uniguttatus*, and *P. antennata*. Numbers on bars represent "the number of positive insect samples/total number of examined insect samples."

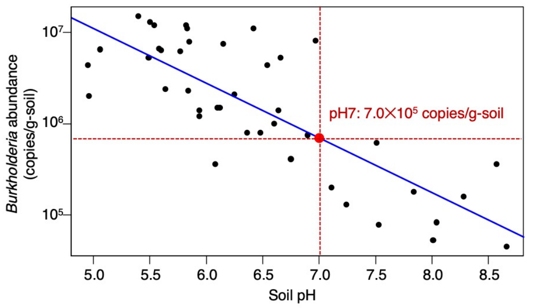

Fig. S6. Relationship between soil pH and *Burkholderia* abundance across 42 soil samples used for the laboratory experiment shown in Fig. 3C. Each point represents an individual soil sample. *Burkholderia* abundance (copies g^{-1} soil) is shown on a log scale. The solid blue line indicates the linear regression fitted to log_{10}-transformed *Burkholderia* abundance. The red point and dashed lines indicate the estimated *Burkholderia* abundance at pH 7.0.

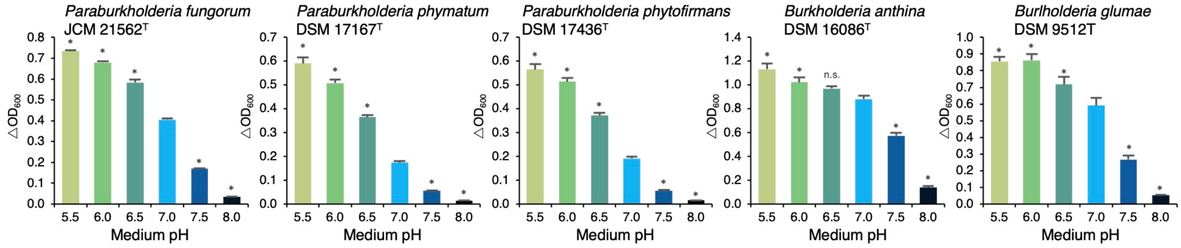

Fig. S7. pH preferences for growth of type strains within *Burkholderia sensu lato*. pH preferences of three *Paraburkholderia* type strains and two *Burkholderia sensu stricto* type strains were checked using media with pH ranging from 5.5 to 8.0. Error bars represent standard deviation (n = 3). Values marked with an asterisk indicate a significant difference compared to those at pH 7.0, as determined by Dunnett's test: * ($P < 0.05$); n.s., $P \geq 0.05$, not significant.

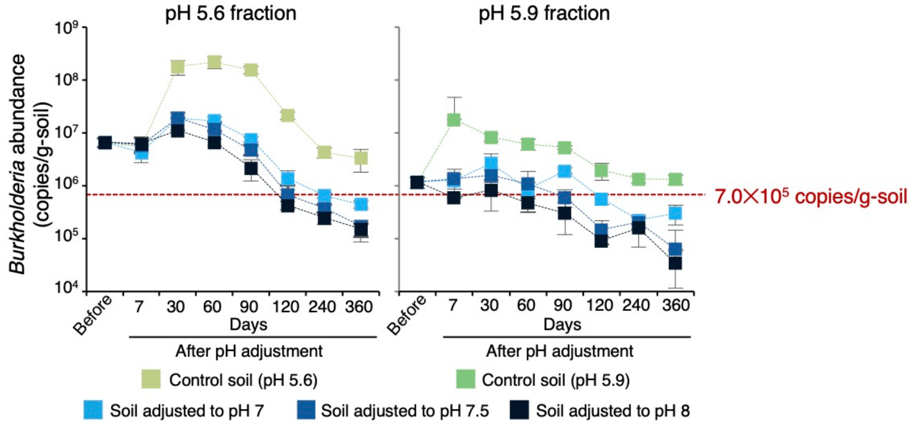

Fig. S8. Transition of *Burkholderia* abundance in soils after pH modification to levels of 7–8 by $CaCO_3$ addition. Error bars represent standard deviation (n = 3). The dashed red lines indicate the threshold required for successful symbiont acquisition, estimated in Fig.S6.

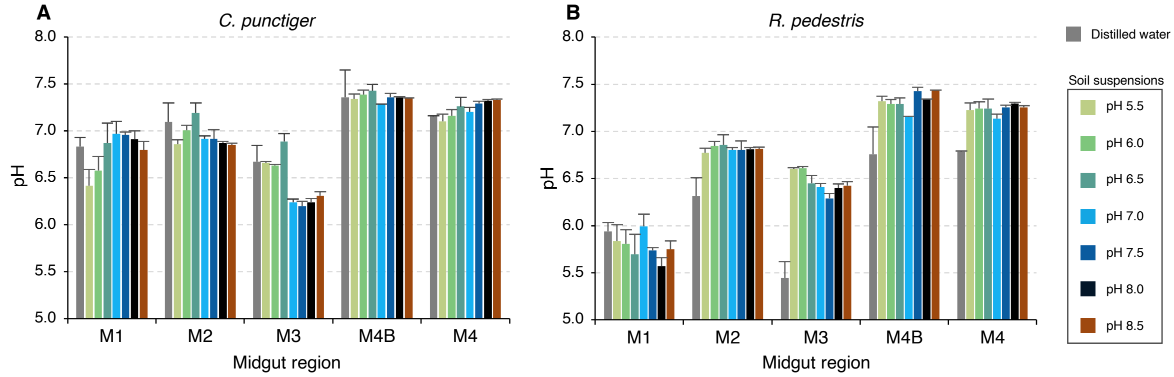

Fig. S9. Gut pH distribution in stink bugs following the oral administration of soil suspensions with various pH levels. One week after the oral administration of distilled water or soil suspensions, the midgut sections of the insects were dissected and collected in distilled water for pH measurement (n = 3). (A) and (B) Data from *C. punctiger* and *R. pedestris*, respectively. Abbreviations: M1, midgut first section; M2, midgut second section; M3, midgut third section; M4B, midgut fourth section with bulb; M4, midgut fourth section with crypts (symbiotic organ); H, hindgut. Error bars represent standard deviation (n = 3).

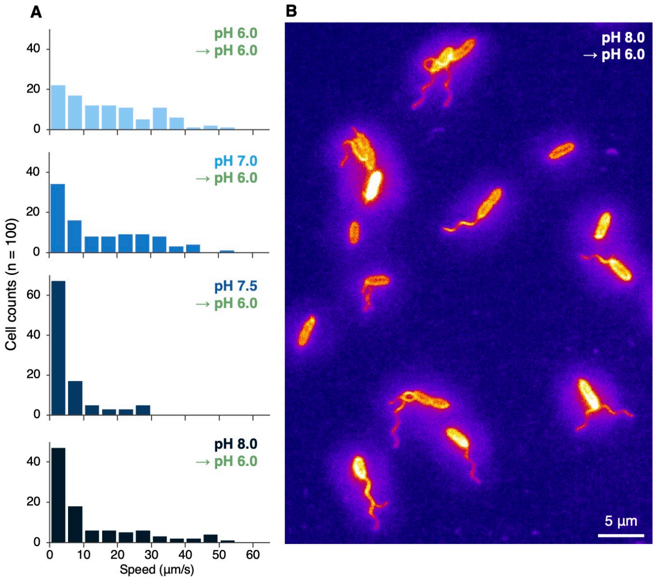

Fig. S10. *In vitro* observation of flagellar motility in symbiotic *Burkholderia* following pH modifications from 7.0 to 8.0 and then back to 6.0. (A) Swimming speed distribution of *Burkholderia* cells (n = 100) 4 h after each pH modification. (B) Fluorescence microscopy images of flagella in *Burkholderia* cells captured 4 h after pH modification from 8.0 to 6.0.

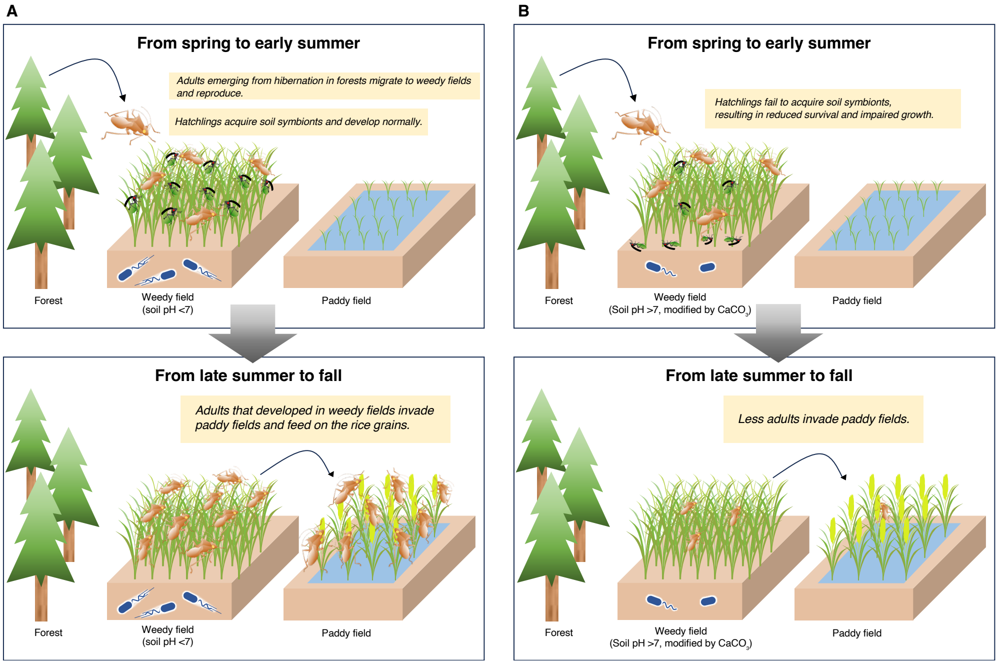

Fig. S11. Life cycle and invasion pathway of rice stink bugs, and the potential effect of soil pH modification in weedy breeding grounds. (A) Schematic illustration showing the seasonal life cycle and invasion pathway of rice stink bugs. In spring, adults emerge from hibernation in forests and migrate to weedy fields. From spring to early summer, they reproduce in weedy fields, and the hatchlings develop normally by acquiring symbionts from the soil. Upon reaching adulthood and developing wings, the adults migrate into rice paddies in late summer to autumn, when rice plants enter the grain-filling stage, and feed on the rice grains. (B) Conceptual illustration of the effect of soil pH modification in weedy fields (soil pH >7 adjusted with $CaCO_3$). Even if overwintered adults lay eggs in these fields, hatched nymphs fail to acquire symbionts from the soil, resulting in reduced survival and impaired growth. Consequently, the number of adults invading rice paddies is reduced.

Table S1. Prevalence of *Burkholderia* among insect samples.

Insect taxa			Infection rate to *Burkholderia*[a]				
Superfamily	Family	Genus Species	Wild population	Eggs	Instars reared without soil and adults	Instars reared with soil[b]	Instars reared with adults
Coreoidea	Coreoidae	*Cletus punctiger*	100% (47/47)	0% (0/32)	0% (31/31)	100% (32/32)	0% (0/30)
		Cletus schmidti	100% (36/36)	0% (0/30)	0% (32/32)	100% (32/32)	51.7% (31/60)
	Alydidae	*Riptortus pedestris*	97.6% (87/89)[c]	0% (0/198)[d]	0% (0/48)	100% (8/8)	0% (0/30)
		Leptocorisa chinensis	98.3% (114/116)[c]	0% (0/32)	0% (0/27)	100% (8/8)	0% (0/32)
Lygaeoidea	Rhyparochromidae	*Metochus uniguttatus*	100% (10/10)	0% (0/24)	0% (0/16)	100% (8/8)	0% (0/30)
	Pachygronthidae	*Pachygrontha antennata*	100% (32/32)	0% (0/32)	0% (0/16)	93.8% (15/16)	0% (0/16)

[a] Values indicate "the number of positive insect samples/total number of examined insect samples."
[b] Reared with soil S32 (Table S5).
[c] Data from the previous study (Kikuchi et al., 2005).
[d] Data from the previous study (Kikuchi et al., 2007).

Table S2. Insect samples analyzed for gut microbiota using PCR amplicon sequencing of the bacterial 16S rRNA gene.

Insect species	Collection site	Dominant plant species at the collection site	Collection date	No. of individuals
Cletus punctiger	Tsukubamirai, Ibaraki	*Digitaria ciliaris / Setaria viridis*	22nd Aug. 2019	1
	Kashiwa, Chiba	*Digitaria ciliaris / Setaria viridis*	22nd Aug. 2019	1
	Yaizu, Shizuoka	*Digitaria ciliaris / Setaria viridis*	2nd Sep. 2019	1
	Chiba, Abiko	*Digitaria ciliaris / Setaria viridis*	22nd Aug. 2019	1
	Hamamatsu, Shizuoka	*Digitaria ciliaris / Setaria viridis*	6th Sep. 2019	1
	Yawatahama, Ehime	*Digitaria ciliaris / Setaria viridis*	2nd Nov. 2015	1
	Takaoka-gun, Kochi	*Digitaria ciliaris / Setaria viridis*	1st Nov. 2015	1
	Nakagawa, Fukuoka	*Digitaria ciliaris / Setaria viridis*	3rd Nov. 2015	1
	Yame, Fukuoka	*Digitaria ciliaris / Setaria viridis*	30th Oct. 2015	1
	Oita, Oita	*Digitaria ciliaris / Setaria viridis*	2nd Nov. 2015	1
	Kanzaki, Saga	*Digitaria ciliaris / Setaria viridis*	29th Oct. 2015	1
	Tamana, Kumamoto	*Digitaria ciliaris / Setaria viridis*	30th Oct. 2015	1
Cletus schmidti	Sapporo, Hokkaido	*Persicaria lapathifolia*	2nd Aug. 2014	1
	Tsukubamirai, Ibaraki	*Persicaria longiseta*	23rd Aug. 2019	1
	Agawa-gun, Kochi	*Achyranthes bidentata*	1st Nov. 2015	1
	Takaoka-gun, Kochi	*Achyranthes bidentata*	1st Nov. 2015	1
	Nishiuwa-gun, Ehime	*Achyranthes bidentata*	2nd Nov. 2015	1
	Oita, Oita	*Persicaria longiseta*	2nd Nov. 2015	1
Riptortus pedestris	Tsukubamirai, Ibaraki	*Glycine max*	22nd Aug. 2019	10
Leptocorisa chinensis	Ishioka, Ibaraki	*Digitaria ciliaris / Setaria viridis*	19th Aug. 2020	20
Metochus uniguttatus	Naha, Okinawa	–[a]	14th Jul. 2019	3
	Naha, Okinawa	–[a]	15th Nov. 2019	7
Pachygrontha antennata	Ishioka, Ibaraki	*Digitaria ciliaris / Setaria viridis*	19th Aug. 2020	10

[a] *M. unigutatus* specimens were collected from fallen leaves in a sparse forest.

Table S3. Details of the statistical analyses for the fitness effects of *Burkholderia* symbiosis in *Cletus* spp. presented in Fig. 2 (provided in a separate Excel sheet).

Table S4. Weedy fields surveyed for soil pH and insect density in this study (provided in a separate Excel sheet).

Table S5. Soil samples used in laboratory experiments (provided in a separate Excel sheet).

Table S6. Results of statistical analyses using Generalized Linear Model (GLM).

Constructed model	Factor	Estimate	95% CI	*P* value	R^2
**Total bacteria vs pH**					
GLM with Gaussian distribution	Intercept	10.084	[9.460, 10.707]	< 0.001	
	pH	−0.062	[−0.158, 0.034]	0.205	
Test of dispersion				0.84	
Coefficient of determination					0.075
**Relative abundance of Burkholderia vs pH**					
GLM with betabinomial distribution	Intercept	−2.929	[−4.578, −1.280]	< 0.001	
	pH	−0.677	[−0.950, −0.403]	< 0.001	
Test of dispersion				0.224	
Coefficient of determination					0.016
***C. punctiger* vs pH**					
GLM with betabinomial distribution	Intercept	21.715	[12.796, 30.634]	< 0.001	
	pH	−3.223	[−4.577, −1.869]	< 0.001	
Test of dispersion				0.768	
Coefficient of determination					0.366
***R. pedestris* vs pH**					
GLM with betabinomial distribution	Intercept	17.64	[11.974, 23.307]	< 0.001	
	pH	−2.590	[−3.441, −1.739]	< 0.001	
Test of dispersion				0.576	
Coefficient of determination					0.312
**Infecion ratio of *C. punctiger* in pH 5.6 fraction**					
GLM with betabinomial distribution	Intercept	13.885	[9.320, 18.451]	< 0.001	
	pH	−2.590	[−2.727, −1.422]	< 0.001	
Test of dispersion				0.464	
Coefficient of determination					0.248
**Infecion ratio of *C. punctiger* in pH 5.9 fraction**					
GLM with betabinomial distribution	Intercept	23.648	[12.858, 34.438]	< 0.001	
	pH	−3.531	[−5.124, −1.939]	< 0.001	
Test of dispersion				0.744	
Coefficient of determination					0.261
**Infecion ratio of *R. pedestris* in pH 5.6 fraction**					
GLM with betabinomial distribution	Intercept	16.071	[10.551, 21.590]	< 0.001	
	pH	−2.240	[−2.988, −1.492]	< 0.001	
Test of dispersion				0.672	
Coefficient of determination					0.304
**Infecion ratio of *R. pedestris* in pH 5.9 fraction**					
GLM with betabinomial distribution	Intercept	16.709	[11.020, 22.399]	< 0.001	
	pH	−3.531	[−3.093, −1.526]	< 0.001	
Test of dispersion				0.624	
Coefficient of determination					0.228
**Burkholderia abundance in pH 5.6 fraction**					
GLM with Gaussian distribution	Intercept	10.039	[8.113, 11.966]	< 0.001	
	pH	−2.240	[−0.755, −0.215]	< 0.001	
Test of dispersion				0.84	
Coefficient of determination					0.145
**Burkholderia abundance in pH 5.9 fraction**					
GLM with Gaussian distribution	Intercept	10.335	[8.750, 11.920]	< 0.001	
	pH	−3.531	[−0.839, −0.401]	< 0.001	
Test of dispersion				0.84	
Coefficient of determination					0.385

Table S7. Correlation between the soil chemical properties and the relative abundance of *Burkholderia* in the soil microbiome, for 42 soils used for the infection experiment.

Soil chemical properties	*P* value	*Q* value	R^2
pH	**0.000003286**	**0.000011949**	0.016
Water content (%)	0.4167	0.13261	0.001
EC (mS/cm)	**0.006474**	**0.0078**	0.005
CEC (meq/100 g)	0.1722	0.0895	0.001
Exchangeable Ca (mg/100 g)	**0.00002495**	**0.000045364**	0.013
Exchangeable Mg (mg/100 g)	0.5677	0.15880	0.000
Exchangeable K (mg/100 g)	0.7744	0.20114	0.000
Available phosphate (mg/100 g)	0.09977	0.06047	0.002
Phosphate absorption coefficient (mg/100 g)	**0.03497**	**0.03179**	0.003
NH_4-N (mg/100 g)	0.2128	0.09673	0.001
NO_3-N (mg/100 g)	0.09874	0.06047	0.002
Humus (%)	0.4364	0.13261	0.000
Total C (g/kg)	0.4376	0.13261	0.000
Total N (g/kg)	0.4139	0.13261	0.001

Table S8. Insect samples analyzed for the prevalence of *Burkholderia* in wild populations using diagnostic PCR.

Insect species	Collection site	Dominat plant species at the collection site	Collection date	No. of individuals
Cletus punctiger	Saga, Kanzaki	*Digitaria ciliaris / Setaria viridis*	29th Oct. 2015	19
	Koshi, Kumamoto	*Digitaria ciliaris / Setaria viridis*	30th Oct. 2014	28
Cletus schmidti	Takaoka-gun, Kochi	*Achyranthes bidentata*	1st Nov. 2015	12
	Nishiuwa-gun, Ehime	*Achyranthes bidentata*	1st Nov. 2015	24
Metochus uniguttatus	Naha, Okinawa	–[a]	14th Jul. 2019	3
	Naha, Okinawa	–[a]	15th Nov. 2019	7
Pachygrontha antennata	Ishioka, Ibaraki	*Digitaria ciliaris / Setaria viridis*	19th Aug. 2020	32

[a] *M. unigutatus* were captured on fallen leaves in a sparse forest.

Movie S1. The *Burkholderia* treated at pH 6.5, moving within the M4B region in the insect gut (provided in a separate movie file). The green signals represent GFP-labeled *Burkholderia*.

Movie S2. The *Burkholderia* treated at pH 7.5, staying within the M3 region in the insect gut (provided in a separate movie file). The green signals represent GFP-labeled *Burkholderia*.

References

Kikuchi Y, Meng X-Y, Fukatsu T., 2005. Gut symbiotic bacteria of the genus *Burkholderia* in the broad-headed bugs *Riptortus clavatus* and *Leptocorisa chinensis* (heteroptera: Alydidae). *Applied and Environmental Microbiology* 71: 4035-43.

Kikuchi Y, Hosokawa T, Fukatsu T., 2007. Insect-microbe mutualism without vertical transmission: A stinkbug acquires a beneficial gut symbiont from the environment every generation. *Applied and environmental microbiology* 73: 4308-16.

Kikuchi, Y., Hosokawa, T., and Fukatsu, T., 2011. An ancient but promiscuous host–symbiont association between Burkholderia gut symbionts and their heteropteran hosts. *ISME Journal* 5: 446-460.

Mullins, A.J. and Mahenthiralingam, E., 2021. The hidden genomic diversity, specialized metabolite capacity, and revised taxonomy of *Burkholderia sensu lato*. *Frontiers in microbiology*, 12: 726847.
